# Supplementary material for: Large-scale simulation of biomembranes incorporating realistic kinetics into coarse-grained models
Source: Nat Commun. 2020 Jun 11;11:2951. doi: 10.1038/s41467-020-16424-0 (PMC7289815; doi:10.1038/s41467-020-16424-0)
Supplement: Supplementary file 1 — Supplementary Information [file 41467_2020_16424_MOESM1_ESM.pdf]

SUPPLEMENTARY INFORMATION

TABLE 1: Properties of the membrane used for force field parametrization [1–3].

d_{m} (nm)	$\kappa\,(kT)$	$\bar{\kappa}\,(kT)$	$K_{\mathrm{area}}(\mathrm{N\,m^{-1}})$
4.0	18.73	-14.98	0.270

TABLE 2: Force field parameters for simulation of planar membranes.

r_{eq} (nm)	D_{e} $(\mathrm{kJ\,mol^{-1}})$	α $(\mathrm{nm^{-1}})$	θ_{eq} (rad)	K_{b} $(\mathrm{kJ\,mol^{-1}})$	d_{eq} (nm)	K_{d} $(\mathrm{kJ\,mol^{-1}\,nm^{-2}})$
10.0	9.91	0.12	$\pi/2$	20.74	4.0	6.19
5.0	14.45	0.20	$\pi/2$	12.38	4.0	6.19

TABLE 3: Properties of the red blood cell [4–7].

d_{m} (nm)	$\kappa\,(kT)$	$\bar{\kappa}\,(kT)$	$K_{\mathrm{area}}(\mathrm{N\,m^{-1}})$	$\mu_{\mathrm{m}}(\mathrm{Pa\,s})$	$\sigma_{\mathrm{m}}(\mathrm{N\,m^{-1}})$	$V_{\mathrm{eq}}(\mathrm{fL})$
4.0	5.6	-4.48	15.5×10^{-6}	0.022	6.5×10^{-7}	107.96

TABLE 4: Similar to Tab. 2, for red blood cell simulations.

r_{eq} (nm)	D_{e} ($\mathrm{kJ\,mol^{-1}}$)	α ($\mathrm{nm^{-1}}$)	θ_{eq} (rad)	K_{b} ($\mathrm{kJ\,mol^{-1}}$)	d_{eq} (nm)	K_{d} ($\mathrm{kJ\,mol^{-1}\,nm^{-2}}$)
20.0	83.08	0.06	$\pi/2$	38.05	4.0	3.09
200.0	9.91	0.006	$\pi/2$	9.30	4.0	15.47

[1] D. Marsh, Chem. Phys. Lipids **144**, 146 (2006).

[2] R. Dimova, Adv. Colloid Interface Sci. **208**, 225 (2014).

[3] E. Chacón, P. Tarazona, and F. Bresme, J. Chem. Phys. **143**, 034706 (2015).

[4] D. A. Fedosov, B. Caswell, and G. E. Karniadakis, Biophys. J. **98**, 2215 (2010).

[5] T. Betz, M. Lenz, J.-F. Joanny, and C. Sykes, Proc. Natl. Acad. Sci. **106**, 15320 (2009).

[6] A. A. Evans, B. Bhaduri, G. Popescu, and A. J. Levine, Proc. Natl. Acad. Sci. **114**, 2865 (2017).

[7] H. Turlier, D. A. Fedosov, B. Audoly, T. Auth, N. S. Gov, C. Sykes, J.-F. Joanny, G. Gompper, and T. Betz, Nat. Phys. **12**, 513 (2016).
