# Supplementary material for: Large-scale simulation of biomembranes incorporating realistic kinetics into coarse-grained models
Source: Nat Commun. 2020 Jun 11;11:2951. doi: 10.1038/s41467-020-16424-0 (PMC7289815; doi:10.1038/s41467-020-16424-0)
Supplement: Supplementary file 3 — Reporting Summary [file 41467_2020_16424_MOESM3_ESM.pdf]

nature research

Corresponding author(s): Mohsen Sadeghi
Frank Noé

Last updated by author(s): Apr 20, 2020

Reporting Summary

Nature Research wishes to improve the reproducibility of the work that we publish. This form provides structure for consistency and transparency in reporting. For further information on Nature Research policies, see Authors & Referees and the Editorial Policy Checklist .

Statistics

For all statistical analyses, confirm that the following items are present in the figure legend, table legend, main text, or Methods section.

n/a	Confirmed	
☐	☒	The exact sample size (*n*) for each experimental group/condition, given as a discrete number and unit of measurement
☐	☒	A statement on whether measurements were taken from distinct samples or whether the same sample was measured repeatedly
☐	☒	The statistical test(s) used AND whether they are one- or two-sided<br>*Only common tests should be described solely by name; describe more complex techniques in the Methods section.*
☒	☐	A description of all covariates tested
☐	☒	A description of any assumptions or corrections, such as tests of normality and adjustment for multiple comparisons
☐	☒	A full description of the statistical parameters including central tendency (e.g. means) or other basic estimates (e.g. regression coefficient) AND variation (e.g. standard deviation) or associated estimates of uncertainty (e.g. confidence intervals)
☒	☐	For null hypothesis testing, the test statistic (e.g. *F*, *t*, *r*) with confidence intervals, effect sizes, degrees of freedom and *P* value noted<br>*Give P values as exact values whenever suitable.*
☒	☐	For Bayesian analysis, information on the choice of priors and Markov chain Monte Carlo settings
☒	☐	For hierarchical and complex designs, identification of the appropriate level for tests and full reporting of outcomes
☒	☐	Estimates of effect sizes (e.g. Cohen's *d*, Pearson's *r*), indicating how they were calculated

Our web collection on statistics for biologists contains articles on many of the points above.

Software and code

Policy information about availability of computer code

Data collection	Calculations based on the developed hydrodynamic method are performed with in-house Python codes available from the public repository referenced in the text.<br>Membrane simulations are performed with a specific-purpose C++ code developed in-house, and freely available according to our Software Availability clause.
Data analysis	NumPy 1.18.1, SciPy 1.4.1<br>Custom codes for processing of trajectories and feeding the data to the modules of packages, implemented in Python as Jupyter notebooks.

For manuscripts utilizing custom algorithms or software that are central to the research but not yet described in published literature, software must be made available to editors/reviewers. We strongly encourage code deposition in a community repository (e.g. GitHub). See the Nature Research guidelines for submitting code & software for further information.

Data

Policy information about availability of data

All manuscripts must include a data availability statement. This statement should provide the following information, where applicable:

- Accession codes, unique identifiers, or web links for publicly available datasets
- A list of figures that have associated raw data
- A description of any restrictions on data availability

The data that support the findings of this study are available from the corresponding authors upon request.

Field-specific reporting

Please select the one below that is the best fit for your research. If you are not sure, read the appropriate sections before making your selection.

☒ Life sciences ☐ Behavioural & social sciences ☐ Ecological, evolutionary & environmental sciences

For a reference copy of the document with all sections, see nature.com/documents/nr-reporting-summary-flat.pdf

Life sciences study design

All studies must disclose on these points even when the disclosure is negative.

Sample size	Analysis based on simulation results have been done with maximum possible sample size, considering the required computing time and memory limitations in the analysis. No systematic reduction of samples has been performed.
Data exclusions	No data exclusion has been done in the selected sections of the trajectories used for each analysis. The trajectory sections have been chosen based on the required relaxation times, and meaningful required sample sizes. The procedure has been described in the text.
Replication	No new experimental findings has been reported in the manuscript. Reproducibility of the simulation results is ensured through clear representation of all the methods used, and publicly shared codes.
Randomization	Where needed, grouping of data has been achieved using randomization.
Blinding	Blinding is not relevant to this study, as the findings are based on a physical model described in the manuscript and the software code developed based on it.

Reporting for specific materials, systems and methods

We require information from authors about some types of materials, experimental systems and methods used in many studies. Here, indicate whether each material, system or method listed is relevant to your study. If you are not sure if a list item applies to your research, read the appropriate section before selecting a response.

Materials & experimental systems

n/a	Involved in the study	
☒	☐	Antibodies
☒	☐	Eukaryotic cell lines
☒	☐	Palaeontology
☒	☐	Animals and other organisms
☒	☐	Human research participants
☒	☐	Clinical data

Methods

n/a	Involved in the study	
☒	☐	ChIP-seq
☒	☐	Flow cytometry
☒	☐	MRI-based neuroimaging
